# Cardiovascular Outcomes and Variability in Plasma Lipid Levels Across Body Mass Index Categories: The ARIC Study

**DOI:** 10.1155/jnme/8858333

**Published:** 2025-07-16

**Authors:** Tianyu Xu, Chang Chen, De-Wei An, Yuanyuan Zhou, Zhongping Yu, Yuzhong Wu, Dexi Wu, Xin He, Jiangui He, Yugang Dong, Jan A. Staessen, Chen Liu, Fang-Fei Wei

**Affiliations:** ^1^Department of Cardiology, The First Affiliated Hospital of Sun Yat-Sen University, Guangzhou, Guangdong, China; ^2^NHC Key Laboratory of Assisted Circulation and Vascular Disease, Sun Yat-Sen University, Guangzhou, Guangdong, China; ^3^Biomedical Science Group, University of Leuven, Leuven, Belgium; ^4^Shanghai Institute of Hypertension, State Key Laboratory of Medical Genomics, Ruijin Hospital, Shanghai Jiaotong University School of Medicine, Shanghai, China

**Keywords:** blood lipid variability, heart failure, mortality, myocardial infarction, obesity

## Abstract

The aim of this study was to investigate associations of cardiovascular outcomes with lipid variability across body mass index categories. We identified 6689 participants (57.1% women) enrolled in the Atherosclerosis Risk In Communities (ARICs) study who had ≥ 3 measurements of total cholesterol (TC) and low-density lipoprotein cholesterol (LDL-C). Cox regression models were used to compute hazard ratios (HRs)-associated heart failure (HF), myocardial infarction (MI), and mortality with 1-SD increase in lipid variability captured by SD and variability independent of the mean (VIM). We also assessed whether adding lipid variability would improve the cardiovascular risk prediction beyond the conventional risk factors. Among 2130 (31.8%) obese patients, 1907 (89.5%) had obesity classes I and II and 223 (10.5%) had obesity class III. In multivariable-adjusted analyses, TC and LDL-C variabilities were significantly (*p* ≤ 0.047) associated with HF in overweight (HRs ranging from 1.10 to 1.17), obesity classes I and II (1.11–1.14), and obesity class III (1.21–1.39). Higher TC and LDL-C variabilities conferred higher risk of MI and mortality in obesity classes I and II (*p* ≤ 0.007). Adding TC–VIM and LDL-C–VIM rather than the lipid level to a conventional risk model significantly improved risk prediction of HF with net reclassification improvement amounting to 8.95% for TC–VIM (*p*=0.006) and 8.09% for LDL-C–VIM (*p*=0.012). Elevated TC and LDL-C variabilities were associated with the increased risk of cardiovascular outcomes, particularly in obesity. Our observations highlight the importance of lipid variability in obesity-associated dyslipidemia.

## 1. Introduction

Dyslipidemias refers to abnormal blood lipid levels and is a well-established risk factor for cardiovascular disease [1, 2]. The prevalence of dyslipidemia increased from 11.9% in 2018 to over 30% in 2023, entailing a heavy burden of cardiovascular disease [3]. Obesity is a body mass index (BMI) of 30 kg/m^2^ [4], has a prevalence of approximately 20% in Western Europe [5], and is associated with dyslipidemia in up to 90% of obese patients in the general population [6, 7] as well as in patients with established cardiovascular disease [8, 9]. Morbid obesity defined as a BMI of 40 kg/m^2^ causes left ventricular remodeling and diastolic dysfunction, heart failure (HF) with preserved ejection fraction, and ultimately premature death [10]. Previous studies indicated that higher BMI intensifies the effects of lipid levels on coronary heart disease, indicating the joint benefits of bodyweight control and dyslipidemia management to minimize the risk of CHD [11]. The investigators of the Atherosclerosis Risk in Communities (ARICs) study demonstrated that BMI was significantly associated with incidence of lipid abnormalities [12]. Lipid variability refers to fluctuations in the blood lipid levels over time and is associated with adverse health outcomes [13–16]. However, the association of cardiovascular outcomes with variability in lipids across BMI categories remains unclear. To address this issue, we ran prospective analysis of the ARIC study [17] to assess the association of incident HF, myocardial infarction, and all-cause mortality with variability in the blood lipids across BMI categories. Furthermore, we assessed whether adding lipid variability would improve the cardiovascular risk prediction beyond the conventional risk factors.

## 2. Materials and Methods

### 2.1. Study Population

The ARIC study is a large-scale, long-term, community-based prospective study that from 1987 until 1989 recruited 15,792 participants from 4 US communities: Jackson, Mississippi; Washington County, Maryland; suburbs of Minneapolis, Minnesota; and Forsyth County, North Carolina. The study design and rationale have been described in detail previously [17]. The Institutional Review Boards of all participating study centers approved the protocol. Participants were repeatedly examined, respectively, in 1987–1989 (visit 1), 1990–1992 (visit 2), 1993–1995 (visit 3), and 1996–1998 (visit 4). Of 11,656 participants who attended ARIC visit 4, we excluded 4967 patients because data on lipid measures or covariables were missing (*n* = 1721), because participants had missing or prevalent HF, myocardial infarction, or died before or at visits 4 (*n* = 553), because participants had lipid measured from less than 3 visits after visit 1 (*n* = 196), because subjects had lipid levels measured in a non-fasting state for 12 h (*n* = 1725), or because they were underweight (*n* = 49) or use of statin (*n* = 723) at visit 4. Thus, the number of participants total to 6689 in the present analysis (Supporting Figure 1).

### 2.2. Measurements

Standardized questionnaires were used to collect information on medical history, lifestyle, and intake of medications. Body weight was measured using a scale that was zeroed daily and calibrated quarterly. BMI was categorized as normal (≥ 18.5–< 25.0 kg/m^2^), overweight (≥ 25.0–< 30.0 kg/m^2^), obesity classes I and II (≥ 30.0–< 40.0 kg/m^2^), and morbid class III obesity (≥ 40 kg/m^2^) [18]. In view of the low prevalence of underweight, for analysis, underweight and normal weight were combined for analysis. Blood pressure was measured with a Hawksley random-zero sphygmomanometer at each visit after participants had rested in the seated position for at least 5 min. The second and third of 3 consecutive readings were averaged for analysis. Hypertension was a blood pressure of ≥ 140 mm·Hg systolic or ≥ 90 mm·Hg diastolic or the self-reported use of antihypertensive drugs. Fasting blood samples were obtained by venipuncture for measurement of plasma glucose and plasma lipids: total cholesterol (TC), high-density lipoprotein cholesterol (HDL-C), and triglycerides, using laboratory methods described elsewhere [19]. Low-density lipoprotein cholesterol (LDL-C) was computed by the Friedewald formula [19]. Variability in the blood lipids was assessed across at least 3 visits by the standard deviation (SD) of the mean and variability independent of the mean (VIM) [20]. The estimated glomerular filtration rate (eGFR) was calculated from serum creatinine using the Chronic Kidney Disease Epidemiology Collaboration Equation [21]. Diabetes mellitus was a self-reported diagnosis made by a physician, a fasting plasma glucose of ≥ 126 mg/dL, or use of antidiabetic drugs.

### 2.3. Clinical Outcomes

Incident HF was the primary outcome and obtained as ICD-9 code 410 [22] in the retrospective surveillance of the hospitals serving the catchment area. The incidence of myocardial infarction and all-cause mortality were established through annual follow-up of the cohort, hospital surveillance, and linkage with the National Death Index.

### 2.4. Statistical Analysis

For database management and statistical analysis, we used SPSS Version 26 (SPSS Inc., Chicago, Illinois) and R Version 4.1.2 (R Foundation, Vienna, Austria). Deviation from the normal distribution was assessed by the Kolmogorov–Smirnov statistic. Means were compared using ANOVA or the large sample *z*-test and proportions by the *χ*^2^ statistic. Pearson correlation coefficients were computed between the level and variability of the plasma lipids. The significance was a 2-sided α-level of ≤ 0.05.

In unadjusted exploration analyses, Kaplan–Meier survival functions were constructed stratified by quartiles of the lipid variability indexes and compared by the log-rank test. Time to an adverse health outcome as function of the variability indices was modeled by proportional hazards regression while adjusting for race (White vs. Black), sex, age, BMI, systolic blood pressure, the baseline level of the plasma level under study, eGFR, hypertension, and diabetes mellitus. Hazard ratios (HRs) with 95% confidence interval (CI) express the risk associated with 1-SD in the lipid variability indexes. The performance of lipid variability in risk stratification was assessed by calculating the integrated discrimination improvement (IDI) and the net reclassification improvement (NRI) [23]. IDI is the difference between the discrimination slopes of the basic model and the basic model extended with the lipid variabilities. The discrimination slope is the difference in predicted probabilities (%) between participants with and without an endpoint. To calculate NRI, we predicted in each participant the 10-year risk of an event from a Cox model with and without lipid variabilities.

## 3. Results

### 3.1. Baseline Characteristics of Participants

**Figure 1:**
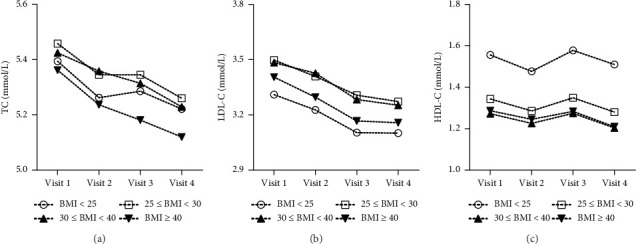
Total cholesterol (a), LDL-C (b), and HDL-C (c) by body mass index (BMI) categories across time points.

Of the 6689 participants retained in the analysis, of whom 2130 (31.8%) were obese, 5674 (84.8%) were Whites, 3819 (57.1%) were women, 2757 (41.2%) had hypertension, and 713 (10.7%) had diabetes at visit 4.  Table 1 shows the characteristics of the participants by categories of BMI. Participants with morbid obesity were more likely to have hypertension and diabetes (Table 1).  Figure 1 indicates the trend of TC, LDL-C, and HDL-C between visit 1 and visit 4. Among participants (37.4%) with normal weight at visit 1, 32.6% became overweight or obese at visit 4 and 27.1% overweight at visit 1 became obese at visit 4 (Supporting Figure 2).

### 3.2. Correlation of the Lipid Variability With Levels

The Pearson correlation coefficients of the lipid levels with variability captured by the SD or VIM ranged from 0.16 to 0.51 and from −0.12 to 0.04, respectively (Supporting Table 1). In within-group comparisons by BMI categories, the correlation coefficients were all significantly greater for SD than for VIM (Supporting Table 1).

### 3.3. HF Related to Lipid Variability

**Figure 2:**
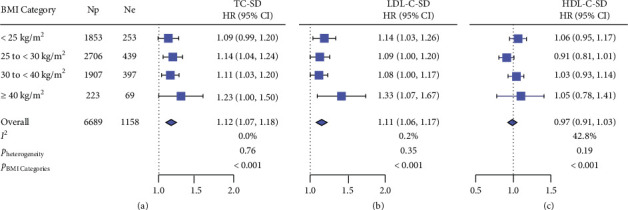
The association of incident HF with SD of total cholesterol (TC) (a), LDL-C (b), and HDL-C (c) stratified by body mass index (BMI) categories. Squares and horizontal lines represent the hazard ratio and 95% confidence interval for each survey cycle. Diamonds denote the pooled estimates with 95% confidence intervals. For *I*^2^, values < 25%, 25%–50%, and > 50% indicate modest, moderate, and substantial heterogeneity, respectively. *p* values refer to the significance of hazard ratios over BMI categories. Np: number of participants at risk and Ne: the number of events.

**Figure 3:**
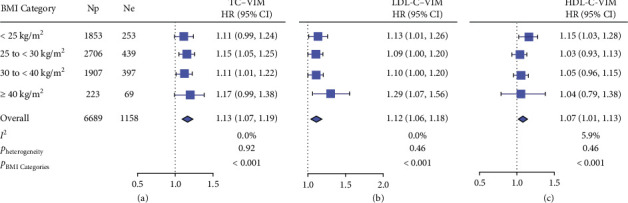
The association of incident HF with VIM of total cholesterol (TC) (a), LDL-C (b), and HDL-C (c) stratified by body mass index (BMI) categories. Squares and horizontal lines represent the hazard ratio and 95% confidence interval for each survey cycle. Diamonds denote the pooled estimates with 95% confidence intervals. For *I*^2^, values < 25%, 25%–50%, and > 50% indicate modest, moderate, and substantial heterogeneity, respectively. *p* values refer to the significance of hazard ratios over BMI categories. Py denotes person-year, Np the number of participants at risk, and Ne the number of deaths.

In crude analyses (Figure 2), the pooled HRs expressing the risk of incident HF associated with a 1-SD increment in lipid variability were 1.12 (95% CI: 1.07–1.18; *p* < 0.001) for TC–SD and 1.11 (95% CI: 1.06–1.17; *p* < 0.001) for LDL-C–SD, respectively. The *p* values for the BMI categories in the HRs were < 0.001 (Figure 2). The corresponding HRs were 1.13 (95% CI: 1.07–1.19; *p* < 0.001) for TC–VIM and 1.12 (95% CI: 1.06–1.18; *p* < 0.001) for LDL-C–VIM, respectively (Figure 3). With adjustments applied for potential confounders, higher TC and LDL-C variabilities were significantly (*p* ≤ 0.047; Table 2) associated with higher risk of HF in participants with overweight (HRs ranging from 1.10 to 1.17), obesity classes I and II (HRs ranging from 1.11 to 1.14), and obesity class III (HRs ranging from 1.21 to 1.39). The HR related the risk of incident HF to LDL-C–SD in morbid obesity was larger (1.39 vs. 1.11) than that in nonmorbid obesity. In White rather than Black, the interaction of morbid obesity with LDL-C–SD was significant for incident HF (*p*=0.009) with adjustments applied for confounders. The analyses (Supporting Table 2) in 7412 participants including those using statins (*n* = 723) and those without using statins (*n* = 6689) produced confirmatory results as shown in Table 2. Adding TC and LDL-C variabilities as calculated by VIM to the reference model, including potential risk factors, increased the risk of HF with NRI amounting to 8.95% (*p*=0.006) and 8.09% (*p*=0.012; Table 3), respectively.

### 3.4. Myocardial Infarction Related to Lipid Variability

In unadjusted analyses (Supporting Figure 3 and Supporting Figure 4), the pooled HRs related incident myocardial infarction to a 1-SD increment in lipid variability were 1.16 (95% CI: 1.08–1.24; *p* < 0.001)/1.11 (1.02–1.21; *p* < 0.001) for TC–SD/TC–VIM and 1.16 (1.08–1.25; *p* < 0.001)/1.11 (1.01–1.22; *p* < 0.001) for LDL-C–SD/LDL-C–VIM, respectively. The *p* values for the BMI categories in the HRs related to variability of TC and LDL-C were ≤ 0.022 (Supporting Figure 3 and Supporting Figure 4). In multivariable-adjusted analyses, higher TC and LDL-C variabilities conferred higher risk of myocardial infarction in obesity classes I and II with HRs ranging from 1.18 to 1.23 (*p* ≤ 0.003; Table 2). Adding TC variability as calculated by SD to the reference model increased the risk of myocardial infarction with NRI amounting to 9.47% (*p*=0.016; Supporting Table 3).

### 3.5. All-Cause Mortality in Relation to Lipid Variability

In crude analyses (Supporting Figure 5 and Supporting Figure 6), the pooled HRs of all-cause mortality associated with a 1-SD lipid variability increment were significant for TC, LDL-C, and HDL-C variabilities with HRs ranging from 1.04 to 1.12 (*p* < 0.001). The *p* values for the BMI categories in the HRs were ≥ 0.084 (Supporting Figure 5 and Supporting Figure 6). In multivariable-adjusted analyses, higher TC, LDL-C, and HDL-C variabilities were stronger predictors of all-cause mortality in all BMI categories with HRs ranging from 1.09 to 1.17 (*p* ≤ 0.007; Table 2) with exception for obesity class III. Adding TC, LDL-C, and HDL-c variabilities as calculated by VIM to the reference model increased the risk of all-cause mortality with IDI ranging from 0.19% to 0.46% (*p* ≤ 0.006; Supporting Table 4) and with NRI ranging from 5.51% to 7.46% (*p* ≤ 0.020; Supporting Table 4).

## 4. Discussion

The key findings of our study can be summarized as follows: (i) higher TC, LDL-C, and HDL-C variabilities conferred higher risk of cardiovascular outcomes, in particular for incident HF and mortality, and (ii) compared with participants with normal weight, higher TC and LDL-C variabilities were associated with increased risk of incident HF, with approximately 10%∼28% increased risk in HF incidence in obesity class III. Our study highlights that variability in blood lipid profiles could be a useful tool to stratify cardiovascular risks in the general population, and more attention should be paid to cardiovascular outcomes with high long-term variability of TC and LDL-C in those with obesity.

A wealth of studies has demonstrated the associations of adverse health outcomes with the variability of lipid levels [13–16, 24, 25]. A single-center cohort study which enrolled 10,583 patients with type 2 diabetes from Taiwan reveals that with each 10% increase in variability of HDL, LDL, and TC, the risk of cardiovascular mortality increased by 27%, 8%, and 16%, respectively [24]. In treating new targets (TNTs) trial of 9572 patients with coronary artery disease, LDL-C variability as calculated by average successive variability was associated with increased risk of myocardial infarction (HR: 1.10; 95% Cl: 1.02–1.19; and *p*=0.02) and mortality (HR for each 1-SD increase, 1.23; 95% Cl: 1.14–1.34; and *p* < 0.001) with adjustments applied for confounders [25]. A meta-analysis of 11 articles from seven cohorts reported that compared with bottom quartile of TC, LDL-C, and HDL-C variabilities, the top quartile conferred high risks of cardiovascular disease and all-cause mortality with HRs ranging from 1.09 to 1.32 [15]. However, those studies did report the effect of obesity (i.e., morbid obesity) on the association of adverse health outcomes with lipid variability. This gap represents a key area of innovation in our research.

Obesity is recognized as a globally epidemic metabolic disease and is viewed as the second major cause of preventable death in Western countries [5]. Obesity is closely associated with cardiovascular risk factors, including dyslipidemias [6, 7, 26], diabetes mellitus [27], and hypertension [28]. Studies have indicated that the prevalence of dyslipidemias incredibly increased in the obese individuals, with an estimated over 2-3-fold increase in the Chinese population [29] and a positive correlation between BMI and LDL-C in the Western population [30]. Nguyen and colleagues demonstrated a remarkable increase in the prevalence of diabetes from 8% in the normal-weight participants to 43% in those with morbid obesity [27]. These studies highlighted the joint benefits of maintaining optimum body weight and blood lipid together with other cardiovascular risk factors.

Several underlying mechanisms may explain why lipid variability conferred increased risk in patients with obesity compared with those with normal weight. First, obesity plays an important role in lipid metabolism characterized by increased triglycerides and subfractions of small, dense LDL, and low HDL-C [31]. The small dense LDL hydrolyze of triglycerides can infiltrate into the subendothelium of the artery and be taken up by macrophages which may lead to endothelial dysfunction in the coronary artery, along with oxidative stress, bringing cholesterol recrystallization and dissolution in atherosclerotic plaque, and finally impair the stability of plaque and the integrity of endothelium and lead atherosclerotic plaque more vulnerable to rupture [32, 33]. Second, morbid obesity is independently associated with the changes of cardiac structure and function, including left ventricular hypertrophy and left ventricular dysfunction [34, 35]. Researchers found that individuals with severe obesity had higher levels of proinflammatory markers such as CRP and IL-6, oxidized LDL, and worse thickness and stiffness in vessels [36]. Finally, morbid obesity is more likely to develop cardiac conduction abnormalities, such as atrial fibrillation [37]. Experimental studies have demonstrated that a marked body weight accumulation resulted in progressive atria remodeling, consisting of a greater fibrosis deposition, higher expression of endothelin receptors, and abnormalities in atrial conduction, leading to easier atrial fibrillation induction [37].

The current study must be interpreted within the context of its merits and potential limitations. To the best of our knowledge, this is the first prospective cohort study to explore the effect of BMI on the association of cardiovascular risk and all-cause mortality with lipid variability. In a relatively large sample size, multivariable-adjusted analyses in which lipid variability was calculated by different metrics yielded robustness of the results. Several of our findings in line with the literature support the validity of our study. Our data indicated an importantly elevated prevalence of dyslipidemia, diabetes, and hypertension in the obese group, particularly in morbid obesity compared with the normal-weight group. Furthermore, we demonstrated that compared with normal weight, obesity class III was associated with an increased risk of incident HF (HR: 2.61 and 95% Cl: 2.00–3.41). However, our study had serval limitations. First, our study could not draw a causal effect of the variability of lipids on the endpoints due to the observational nature. Second, we lack information on stable coronary disease, cerebrovascular disease, and nonalcoholic fatty liver disease, which may influence the association of lipid variability with the risk of outcomes. Third, previous studies suggest that various doses of a statin [25] and different types of statins [38] have different effects on lipid variability. Statin nonadherence is positively associated with great lipid variability [39]. Regarding limited number of participants on statin therapy including only 18 (0.2%) with morbid obesity, we fail to investigate the effect of statin and statin-related adherence on the association of cardiovascular risk with lipid variability across BMI categories. However, our current study found that compared with participants without statin therapy, those taking statin treatment had lower levels of TC and LDL-C but higher levels of lipid variability as reflected by SD and VIM in the new Supporting Table 5. In our study, statin therapy may significantly reduce levels of TC and LDL-C, which lead to greater variability in TC and LDL-C. Thus, with the recommended wide use of statin, it is also called more focus on the fluctuation of lipid levels, which may in a part reflect the consistence and adherence to the therapy rather than the one-time absolute lipid levels to predict cardiovascular events [40]. Finally, our current findings were mainly in the Western population which may be unable to be extrapolated to Chinese and other ethnicities.

## 5. Conclusions

Long-term lipid variability could predict cardiovascular events in the general population free from the history of myocardial infarction and HF. In individuals with morbid obesity, higher variability of lipid profile can provide incremental prognostic value for incident HF, in particular in White. From a clinical perspective, our observations highlight the importance of taking lipid variability as modifiable risk factor to reduce the cardiovascular risk, particularly in those with obesity.

## Figures and Tables

**Table 1:** Baseline characteristics of participants at visit 4 by BMI categories.

Characteristic	BMI categories (kg/m^2^)				*p* value
	BMI < 25	25 ≤ BMI < 30	30 ≤ BMI < 40	BMI ≥ 40	
Number (%) with characteristics	1853 (27.7)	2706 (40.5)	1907 (28.5)	223 (3.33)	
White race	1685 (90.9)	2323 (85.6)^‡^	1511 (79.2)^‡^	155 (69.5)^‡^	< 0.001
Education					
Basic	215 (11.6)	418 (15.5)	344 (18.1)	58 (26.0)	< 0.001
Intermediate	839 (45.3)	1160 (42.9)	835 (43.8)	99 (44.4)	
College or equivalent	797 (43.1)	1126 (41.6)	727 (38.1)	66 (29.6)	
Current smokers	354 (19.1)	321 (11.9)^‡^	171 (9.0)^‡^	15 (6.7)^‡^	< 0.001
Hypertension	555 (30.0)	1074 (39.7)^‡^	987 (51.8)^‡^	141 (63.2)^‡^	< 0.001
Diabetes mellitus	68 (3.67)	256 (9.46)^‡^	333 (17.5)^‡^	56 (25.1)^‡^	< 0.001
Coronary heart disease	21 (1.14)	65 (2.42)^†^	40 (2.11)	1 (0.46)	0.004
Antihypertensive medication	442 (23.9)	885 (32.7)^‡^	883 (46.3)^‡^	124 (55.6)^‡^	< 0.001

*Mean (±SD) of characteristic*					
Age (years)	63.1 ± 5.75	62.9 ± 5.74	62.5 ± 5.43^†^	61.0 ± 5.39^‡^	< 0.001
BMI (kg/m^2^)	22.9 ± 1.58	27.4 ± 1.4^‡^	33.3 ± 2.57^‡^	44.3 ± 3.72^‡^	< 0.001
SBP (mm·Hg)	122.4 ± 18.9	126.4 ± 18.0^‡^	129.5 ± 17.4^‡^	132.3 ± 17.9^‡^	< 0.001
DBP (mm·Hg)	68.5 ± 9.80	71.0 ± 9.65^‡^	72.5 ± 9.85^‡^	72.5 ± 9.58^‡^	< 0.001
Total cholesterol (mmol/L)	5.22 ± 0.92	5.26 ± 0.93^‡^	5.23 ± 0.97^‡^	5.12 ± 0.92	0.13
HDL-C (mmol/L)	1.51 ± 0.47	1.28 ± 0.40^‡^	1.20 ± 0.38^‡^	1.21 ± 0.34^‡^	< 0.001
LDL-C (mmol/L)	3.10 ± 0.86	3.27 ± 0.84	3.25 ± 0.86	3.16 ± 0.82	< 0.001
Triglycerides (mmol/L)	1.33 ± 0.71	1.56 ± 0.82^‡^	1.71 ± 0.85^‡^	1.66 ± 0.82^‡^	< 0.001
eGFR (mL/min/1.73 m^2^)	86.4 ± 14.0	85.6 ± 14.5	86.1 ± 15.8	90.8 ± 16.3^‡^	< 0.001

*Note:* eGFR, glomerular filtration rate estimated from serum creatinine using the Chronic Kidney Disease Epidemiology Collaboration Equation. Significance of the difference with the group with BMI < 25 kg/m^2^: ^†^*p* ≤ 0.01 and ^‡^*p* ≤ 0.001.

Abbreviations: BMI, body mass index; DBP, diastolic blood pressure; HDL-C, high-density lipoprotein cholesterol; LDL-C, low-density lipoprotein cholesterol; SBP, systolic blood pressure.

**Table 2:** Adverse health outcomes associated with lipid variability in BMI categories.

Variability	Body mass index categories (kg/m^2^)			
	BMI < 25	25 ≤ BMI < 30	30 ≤ BMI < 40	BMI ≥ 40
Heart failure				
TC–SD	1.06 (0.96–1.18)	1.17 (1.07–1.28)^‡^	1.14 (1.04–1.24)^†^	1.27 (1.02–1.59)^∗^
TC–VIM	1.10 (0.98–1.22)	1.17 (1.07–1.28)^‡^	1.12 (1.02–1.23)^∗^	1.21 (1.00–1.45)^∗^
LDL-C–SD	1.11 (0.99–1.24)	1.10 (1.00–1.21)^∗^	1.11 (1.02–1.21)^∗^	1.39 (1.09–1.77)^†^
LDL-C–VIM	1.13 (1.01–1.26)^∗^	1.11 (1.01–1.22)^∗^	1.11 (1.01–1.22)^∗^	1.32 (1.07–1.62)^†^
HDL-C–SD	1.14 (1.03–1.27)^∗^	1.01 (0.90–1.13)	1.07 (0.96–1.19)	1.05 (0.77–1.44)
HDL-C–VIM	1.17 (1.05–1.30)^†^	1.05 (0.95–1.15)	1.05 (0.96–1.15)	1.05 (0.79–1.40)
Myocardial infarction				
TC–SD	1.09 (0.96–1.24)	1.08 (0.95–1.23)	1.22 (1.09–1.36)^‡^	1.22 (0.80–1.86)
TC–VIM	1.11 (0.96–1.29)	1.06 (0.93–1.20)	1.23 (1.08–1.40)^†^	1.10 (0.75–1.63)
LDL-C–SD	1.18 (1.05–1.33)^†^	1.07 (0.95–1.21)	1.18 (1.06–1.31)^†^	1.18 (0.73–1.92)
LDL-C–VIM	1.22 (1.07–1.40)^†^	1.07 (0.95–1.22)	1.21 (1.06–1.37)^†^	1.05 (0.64–1.71)
HDL-C–SD	1.20 (1.04–1.40)^∗^	0.93 (0.79–1.10)	1.05 (0.88–1.24)	0.91 (0.46–1.79)
HDL-C–VIM	1.24 (1.07–1.43)^†^	0.95 (0.83–1.08)	1.07 (0.93–1.23)	0.95 (0.55–1.63)
Total mortality				
TC–SD	1.11 (1.04–1.18)^‡^	1.15 (1.08–1.23)^‡^	1.13 (1.06–1.20)^‡^	1.10 (0.89–1.35)
TC–VIM	1.13 (1.06–1.20)^‡^	1.15 (1.08–1.22)^‡^	1.12 (1.05–1.20)^‡^	1.08 (0.91–1.28)
LDL-C–SD	1.14 (1.06–1.21)^‡^	1.09 (1.03–1.16)^†^	1.11 (1.04–1.18)^†^	1.14 (0.91–1.43)
LDL-C–VIM	1.14 (1.06–1.22)^‡^	1.10 (1.03–1.17)^†^	1.11 (1.04–1.19)^†^	1.12 (0.92–1.37)
HDL-C–SD	1.11 (1.04–1.19)^†^	1.17 (1.10–1.25)^‡^	1.11 (1.03–1.20)^†^	1.10 (0.83–1.46)
HDL-C–VIM	1.14 (1.07–1.22)^‡^	1.17 (1.11–1.25)^‡^	1.10 (1.04–1.18)^†^	1.09 (0.85–1.38)

*Note:* Estimates (95% confidence interval) express the hazard ratios of incident heart failure, incident myocardial infarction, and all-cause mortality associated with variability of total cholesterol (TC), LDL-C, and HDL-C, respectively. Adjusted models were adjusted for sex, and race, baseline age, body mass index, systolic blood pressure, corresponding lipid measurement, estimated glomerular filtration rate, hypertension, and diabetes mellitus. Significance of the associations: ^∗^*p* ≤ 0.05, ^†^*p* ≤ 0.01, and ^‡^*p* ≤ 0.001.

**Table 3:** Reclassification and discrimination statistics for association of incident HF with lipid variability.

Models	IDI		NRI	
	Estimate (95% CI) (%)	*p* value	Estimate (95% CI) (%)	*p* value
Total cholesterol				
Basic model	Ref		Ref	
+Baseline level	0.05 (−0.01–0.21)	0.18	5.49 (−3.96–10.23)	0.14
+SD	0.06 (−0.06–0.27)	0.38	8.57 (2.41–13.13)	0.012
+VIM	0.12 (−0.03–0.36)	0.16	8.95 (3.66–14.01)	0.006
+Baseline level + SD	0.15 (−0.02–0.44)	0.096	8.44 (2.73–14.47)	0.004
+Baseline level + VIM	0.17 (−0.02–0.46)	0.084	8.14 (2.92–13.80)	0.006
LDL-C				
Basic model	Ref		Ref	
+Baseline level	0.06 (−0.02–0.22)	0.17	3.81 (−3.80–8.75)	0.25
+SD	−0.01 (−0.09–0.12)	0.92	4.59 (−0.89–10.28)	0.11
+VIM	0.03 (−0.09–0.18)	0.72	8.09 (1.23–12.15)	0.012
+Baseline level + SD	0.08 (−0.07–0.29)	0.34	10.08 (2.74–14.30)	0.016
+Baseline level + VIM	0.08 (−0.07–0.30)	0.34	10.07 (3.63–14.98)	0.012
HDL-C				
Basic model	Ref		Ref	
+Baseline level	0.00 (−0.03–0.10)	0.66	5.30 (−8.27–9.74)	0.80
+SD	0.01 (−0.05–0.13)	0.75	−0.25 (−7.22 to 5.43)	0.97
+VIM	0.05 (−0.04–0.23)	0.29	4.73 (−1.93–9.17)	0.15
+Baseline level + SD	0.03 (−0.04–0.18)	0.34	3.51 (−4.45–9.21)	0.43
+Baseline level + VIM	0.05 (−0.04–0.24)	0.25	4.00 (−2.72–9.96)	0.20

*Note:* The basic model included sex, race, baseline age, body mass index, systolic blood pressure, estimated glomerular filtration rate, hypertension, and diabetes mellitus.

Abbreviations: HDL-C, high-density lipoprotein cholesterol; LDL-C, low-density lipoprotein cholesterol.

## Data Availability

The requests to access the dataset should be sent to the ARIC Data Coordinating Center.

## Supporting Information

The supporting information associated with this study provide detailed background information supporting the methodology and results.

Expanded methods illustrate the equation used to calculate SD and VIM of indices of lipid variability.

Supporting Table 1 presents the correlation coefficients of lipid levels with indices of lipid variability by BMI category.

Supporting Table 2 describes the adverse health outcomes associated with lipid variability in BMI categories in participants using statins and without using statins.

Supporting Tables 3 and 4 report the reclassification and discrimination statistics for association of myocardial infarction and mortality with lipid variability.

Supporting Table 5 summarizes the lipid profile in participants with and without statin therapy.

Supporting Figure 1 presents the flowchart of study design.

Supporting Figure 2 shows the change in BMI categories between ARIC visit 1 and visit 4.

Supporting Figures 3 and 4 describe the association of incident myocardial infarction with SD and VIM of TC, LDL-C, and HDL-C stratified by BMI category.

Supporting Figures 5 and 6 reports the association of all-cause mortality with SD and VIM of TC, LDL-C, and HDL-C stratified by BMI category.

All supporting tables and figures are referenced in the manuscript and support the conclusions drawn in the statistical analysis.
