# Supplementary material for: Cardiovascular Outcomes and Variability in Plasma Lipid Levels Across Body Mass Index Categories: The ARIC Study
Source: J Nutr Metab. 2025 Jul 16;2025:8858333. doi: 10.1155/jnme/8858333 (PMC12286673; doi:10.1155/jnme/8858333)
Supplement: Supporting Information — Additional supporting information can be found online in the Supporting Information section. [file 8858333.f1.doc]

**Cardiovascular Outcomes and Variability in Plasma Lipid Levels
Across Body Mass Index Categories: The ARIC Study**

Tianyu Xu,^1,2*^ Chang Chen,^1,2*^ De-Wei An,^3,4^ Yuanyuan Zhou,^1,2^ Zhongping Yu,^1,2^
Yuzhong Wu,^1,2^ Dexi Wu,^1,2^ Xin He,^1,2^ Jiangui He,^1,2^ Yugang Dong,^1,2^
Jan A. Staessen,^3^ Chen Liu,^1,2†^ and Fang-Fei Wei^1,2,†^

**Author Affiliations**

**:**
^1^Department of Cardiology, the First Affiliated Hospital of Sun Yat-Sen University, Guangzhou, Guangdong, China; ^2^NHC Key Laboratory of Assisted Circulation and Vascular Disease, Sun Yat‑Sen University, Guangzhou, Guangdong, China; ^3^Biomedical Science Group, University of Leuven, Leuven, Belgium; ^4^Shanghai Institute of Hypertension, State Key Laboratory of Medical Genomics, Ruijin Hospital, Shanghai Jiaotong University School of Medicine, Shanghai, China.

* Joint first authors who contributed equally.

† Joint senior authors who contributed equally.

**Table of Contents**

| Expanded method for SD and VIM of lipid profile | p3 |
| --- | --- |
| Supplementary TABLE 1: The correlation coefficients of lipid levels with indexes of lipid variability by   BMI category. | p4 |
| Supplementary TABLE 2:  Adverse health outcomes associated with lipid variability in BMI categories   in participants using statins (n=723) and without using statins (n=6689) | p5 |
| Supplementary TABLE 3: Reclassification and discrimination statistics for association of myocardial  infarction with lipid variability. | p6 |
| Supplementary TABLE 4: Reclassification and discrimination statistics for association of mortality   with lipid variability. | p7 |
| Supplementary TABLE 5: Lipid profile in participants with and without statin therapy. | p8 |
| Supplementary FIGURE 1: Flow chart of study design. | p9 |
| Supplementary FIGURE 2: Change in body mass index categories between ARIC visit 1 and visit 4. | p10 |
| Supplementary FIGURE 3: The association of incident myocardial infarction with SD of total   cholesterol, LDL‑C and HDL‑C stratified by body mass index category. | p11 |
| Supplementary FIGURE 4: The association of incident myocardial infarction with VIM of total   cholesterol, LDL‑C and HDL‑C stratified by body mass index category. | p12 |
| Supplementary FIGURE 5: The association of all‑cause mortality with SD of total cholesterol, LDL‑C  and HDL‑C stratified by body mass index category | p13 |
| Supplementary FIGURE 6: The association of all‑cause mortality with VIM of total cholesterol, LDL‑C   and HDL‑C stratified by body mass index category | p14 |

**Expanded Methods**

The standard deviation (SD) was calculated as shown:

**SD = sqrt [ Σ(X_i_ – μ)2 / N ]**,

in which Σ = summation, X_i_ = each value, μ = the mean of the set of values, N = total number of values.

VIM is the within subject SD divided by the within-subject mean blood pressure level to the power *x* and multiplied by the population mean blood pressure level to the power *x* [1,2]. The power *x* is obtained by fitting a curve through a plot of SD against mean blood pressure level, using the model SD=a × mean*^x^*, where *x* is derived by nonlinear regression. The VIM was calculated as **VIM = (SD / mean^x^)**.

**References**

1. Rothwell PM, Howard SC, Dolan E, et al. Effects of beta blockers and calcium-channel blockers on within-individual variability in blood pressure and risk of stroke. *Lancet Neurol*. 2010;9:469–480.
2. Rothwell PM, Howard SC, Dolan E, et al. Prognostic significance of visit-to-visit variability, maximum systolic blood pressure, and episodic hypertension. *Lancet*. 2010;375:895–905.

Supplementary TABLE 1: Correlation coefficients of lipid levels with indices of lipid variability
by BMI category.

| **Variability Model** | BMI<25 | **25≤BMI<30** | **30≤BMI<40** | **BMI≥40** |
| --- | --- | --- | --- | --- |
| TC and variability |  |  |  |  |
| SD | 0.33‡ | 0.28‡ | 0.33‡ | 0.21† |
| VIM | -0.02 | -0.09‡ | -0.01 | -0.11 |
| LDL-C and variability |  |  |  |  |
| SD | 0.28‡ | 0.24‡ | 0.28‡ | 0.16* |
| VIM | -0.05* | -0.05* | 0.01 | -0.12 |
| HDL-C and variability |  |  |  |  |
| SD | 0.46‡ | 0.49‡ | 0.51‡ | 0.49‡ |
| VIM | -0.02 | 0.01 | 0.04 | 0.04 |

Significance of the correlation coefficients: * *p* ≤ 0.05, † *p* ≤ 0.01, and ‡ *p* ≤ 0.001.

Supplementary TABLE 2:

Adverse health outcomes associated with lipid variability in BMI categories in
 participants using statins (n=723) and without using statins (n=6689).

| **Variability** | **Body mass index categories, kg/m²** | | | |
| --- | --- | --- | --- | --- |
|  | **BMI<25** | **25≤BMI<30** | **30≤BMI<40** | **BMI≥40** |
| **Heart failure** | | | | |
| TC-SD | 1.10 (0.99-1.22) | 1.16 (1.06-1.28)† | 1.16 (1.06-1.26)† | 1.37 (1.09-1.71)† |
| TC-VIM | 1.13 (1.01-1.26)* | 1.15 (1.05-1.26)† | 1.14 (1.04-1.25)† | 1.25 (1.04-1.50)* |
| LDL-C-SD | 1.14 (1.01-1.28)* | 1.11 (1.01-1.22)* | 1.12 (1.03-1.22)* | 1.53 (1.21-1.94)‡ |
| LDL-C-VIM | 1.15 (1.03-1.29)* | 1.11 (1.01-1.22)* | 1.11 (1.01-1.22)* | 1.39 (1.14-1.70)† |
| HDL-C-SD | 1.14 (1.03-1.26)* | 1.00 (0.90-1.12) | 1.07 (0.97-1.18) | 1.07 (0.80-1.45) |
| HDL-C-VIM | 1.16 (1.05-1.29)† | 1.04 (0.95-1.13) | 1.07 (0.98-1.16) | 1.09 (0.84-1.42) |
| **Myocardial infarction** | | | | |
| TC-SD | 1.14 (1.01-1.29)* | 1.15 (1.02-1.29)* | 1.23 (1.10-1.37)‡ | 1.24 (0.83-1.87) |
| TC-VIM | 1.16 (1.01-1.34)* | 1.12 (0.99-1.26) | 1.24 (1.09-1.40)‡ | 1.11 (0.77-1.60) |
| LDL-C-SD | 1.25 (1.10-1.41)‡ | 1.10 (0.98-1.24) | 1.21 (1.08-1.35)‡ | 1.26 (0.81-1.95) |
| LDL-C-VIM | 1.27 (1.10-1.47)† | 1.11 (0.98-1.25) | 1.24 (1.10-1.41)‡ | 1.14 (0.74-1.77) |
| HDL-C-SD | 1.18 (1.02-1.37)* | 0.96 (0.83-1.11) | 1.06 (0.91-1.24) | 0.71 (0.36-1.40) |
| HDL-C-VIM | 1.20 (1.05-1.39)* | 0.99 (0.88-1.11) | 1.08 (0.96-1.22) | 0.77 (0.45-1.29) |
| **Total mortality** | | | | |
| TC-SD | 1.11 (1.05-1.19)‡ | 1.13 (1.06-1.21)‡ | 1.15 (1.07-1.22)‡ | 1.14 (0.91-1.41) |
| TC-VIM | 1.13 (1.05-1.21)‡ | 1.13 (1.06-1.20)‡ | 1.14 (1.07-1.22)‡ | 1.10 (0.92-1.31) |
| LDL-C-SD | 1.15 (1.07-1.24)‡ | 1.08 (1.01-1.15)* | 1.13 (1.06-1.20)‡ | 1.14 (0.91-1.43) |
| LDL-C-VIM | 1.14 (1.06-1.22)‡ | 1.08 (1.02-1.16)* | 1.13 (1.06-1.21)‡ | 1.14 (0.93-1.40) |
| HDL-C-SD | 1.10 (1.03-1.18)† | 1.17 (1.10-1.25)‡ | 1.09 (1.01-1.17)* | 1.12 (0.86-1.46) |
| HDL-C-VIM | 1.13 (1.05-1.20)‡ | 1.16 (1.10-1.23)‡ | 1.09 (1.02-1.15)† | 1.13 (0.90-1.43) |

Estimates (95% confidence interval) express the hazard ratios of incident heart failure, incident myocardial infarction, and all-cause mortality associated with variability of total cholesterol (TC), LDL-C, and HDL-C, respectively. Adjusted models were adjusted for sex, and race, baseline age, body mass index, systolic blood pressure, corresponding lipid measurement, use of statins, estimated glomerular filtration rate, hypertension, and diabetes mellitus. Significance of the associations: * *p* ≤ 0.05, † *p* ≤ 0.01, and ‡ *p* ≤ 0.001.

Supplementary TABLE 3: Reclassification and discrimination statistics for association of myocardial infarction with lipid variability.

| Models | **IDI** | | **NRI** | |
| --- | --- | --- | --- | --- |
|  | Estimate (95% CI), % | *p* value | Estimate (95% CI), % | *p* value |
| Total cholesterol |  |  |  |  |
| Basic model | ref |  | ref |  |
| + Baseline level | 0.19 (0.03 to 0.48) | 0.008 | 6.05 (0.00 to 12.56) | 0.050 |
| + SD | 0.14 (-0.01 to 0.51) | 0.074 | 9.47 (1.86 to 14.91) | 0.016 |
| + VIM | 0.05 (-0.02 to 0.29) | 0.22 | 4.32 (-2.52 to 10.85) | 0.21 |
| + Baseline level + SD | 0.27 (0.08 to 0.66) | 0.002 | 11.68 (5.45 to 18.45) | <0.001 |
| + Baseline level + VIM | 0.26 (0.08 to 0.63) | <0.001 | 10.28 (4.42 to 16.33) | <0.001 |
| LDL-C |  |  |  |  |
| Basic model | ref |  | ref |  |
| + Baseline level | 0.24 (0.06 to 0.55) | 0.002 | 8.61 (2.55 to 14.48) | 0.004 |
| + SD | 0.10 (-0.03 to 0.32) | 0.18 | 4.41 (-1.55 to 11.06) | 0.17 |
| + VIM | 0.04 (-0.04 to 0.24) | 0.41 | 2.10 (-4.55 to 7.83) | 0.60 |
| + Baseline level + SD | 0.29 (0.08 to 0.63) | <0.001 | 13.91 (5.80 to 18.76) | <0.001 |
| + Baseline level + VIM | 0.30 (0.09 to 0.65) | 0.002 | 13.00 (5.58 to 17.75) | <0.001 |
| HDL-C |  |  |  |  |
| Basic model | ref |  | ref |  |
| + Baseline level | 0.42 (0.20 to 0.77) | <0.001 | 13.89 (7.88 to 19.51) | <0.001 |
| + SD | 0.02 (-0.01 to 0.11) | 0.30 | 7.28 (-10.15 to 12.94) | 0.35 |
| + VIM | 0.02 (-0.02 to 0.14) | 0.50 | -3.03 (-8.33 to 3.99) | 0.40 |
| + Baseline level + SD | 0.43 (0.21 to 0.80) | <0.001 | 14.45 (8.85 to 21.09) | <0.001 |
| + Baseline level + VIM | 0.44 (0.21 to 0.80) | <0.001 | 15.41 (9.64 to 21.75) | <0.001 |

LDL-C denotes low-density lipoprotein cholesterol and HDL-C high-density lipoprotein cholesterol. The basic model included sex, and race, baseline age, body mass index, systolic blood pressure, estimated glomerular filtration rate, hypertension, and diabetes mellitus.

Supplementary TABLE 4: Reclassification and discrimination statistics for association of mortality with lipid variability.

| Models | **IDI** | | | **NRI** | |
| --- | --- | --- | --- | --- | --- |
|  | Estimate (95% CI), % | *p* value | Estimate (95% CI), % | | *p* value |
| Total cholesterol |  |  |  | |  |
| Basic model | ref |  | ref | |  |
| + Baseline level | 0.12 (0.01 to 0.30) | 0.042 | 4.93 (-1.21 to 9.14) | | 0.062 |
| + SD | 0.16 (0.02 to 0.36) | 0.022 | 4.84 (0.65 to 9.09) | | 0.032 |
| + VIM | 0.27 (0.09 to 0.54) | <0.001 | 5.51 (0.71 to 9.30) | | 0.020 |
| + Baseline level + SD | 0.35 (0.13 to 0.67) | <0.001 | 7.62 (2.17 to 12.08) | | 0.004 |
| + Baseline level + VIM | 0.34 (0.13 to 0.68) | <0.001 | 6.78 (1.97 to 11.42) | | 0.008 |
| LDL-C |  |  |  | |  |
| Basic model | ref |  | ref | |  |
| + Baseline level | 0.17 (0.03 to 0.37) | 0.010 | 5.62 (0.57 to 9.04) | | 0.030 |
| + SD | 0.09 (-0.03 to 0.26) | 0.16 | 6.83 (1.62 to 10.63) | | 0.012 |
| + VIM | 0.19 (0.03 to 0.40) | 0.006 | 6.08 (1.72 to 10.18) | | 0.006 |
| + Baseline level + SD | 0.30 (0.10 to 0.56) | <0.001 | 9.88 (5.13 to 14.46) | | <0.001 |
| + Baseline level + VIM | 0.30 (0.10 to 0.58) | <0.001 | 10.19 (4.48 to 15.01) | | <0.001 |
| HDL-C |  |  |  | |  |
| Basic model | ref |  | ref | |  |
| + Baseline level | 0.02 (-0.02 to 0.12) | 0.44 | 0.97 (-4.02 to 5.37) | | 0.63 |
| + SD | 0.34 (0.15 to 0.62) | <0.001 | 6.53 (2.62 to 10.73) | | 0.002 |
| + VIM | 0.46 (0.23 to 0.81) | <0.001 | 7.46 (3.83 to 11.78) | | <0.001 |
| + Baseline level + SD | 0.34 (0.15 to 0.64) | <0.001 | 6.83 (2.67 to 10.59) | | 0.002 |
| + Baseline level + VIM | 0.49 (0.25 to 0.86) | <0.001 | 8.11 (4.06 to 12.26) | | <0.001 |

LDL-C denotes low-density lipoprotein cholesterol and HDL-C high-density lipoprotein cholesterol. The basic model included sex, and race, baseline age, body mass index, systolic blood pressure, estimated glomerular filtration rate, hypertension, and diabetes mellitus.

Supplementary TABLE 5: Lipid profile in participants with and without statin therapy.

| **Characteristics** | Statins (n=723) | **Non-statins (n=6689)** | ***p* value** |
| --- | --- | --- | --- |
| BMI categories |  |  | < 0.001 |
| BMI<25 kg/m² | 147 (20.3) | 1853 (27.7) |  |
| 25≤BMI<30 kg/m² | 315 (43.6) | 2706 (40.5) |  |
| 30≤BMI<40 kg/m² | 243 (33.6) | 1907 (28.5) |  |
| BMI≥40 kg/m² | 18 (2.49) | 223 (3.33) |  |
| Mean (±SD) |  |  |  |
| TC, mmol/L | 5.12±0.87 | 5.24±0.94 | 0.002 |
| LDL-C, mmol/L | 3.03±0.76 | 3.21±0.85 | < 0.001 |
| HDL-C, mmol/L | 1.23±0.34 | 1.32±0.43 | < 0.001 |
| TC-SD | 0.85±0.36 | 0.46±0.28 | < 0.001 |
| TC-VIM | 0.74±0.31 | 0.47±0.26 | < 0.001 |
| LDL-C-SD | 0.83±0.36 | 0.44±0.26 | < 0.001 |
| LDL-C-VIM | 0.74±0.31 | 0.45±0.26 | < 0.001 |
| HDL-C-SD | 0.16±0.09 | 0.16±0.11 | 0.093 |
| HDL-C-VIM | 0.17±0.10 | 0.16±0.09 | 0.002 |

Abbreviations: BMI, body mass index; HDL-C, high-density lipoprotein cholesterol; LDL-C, low-density lipoprotein cholesterol; and TC, total cholesterol.


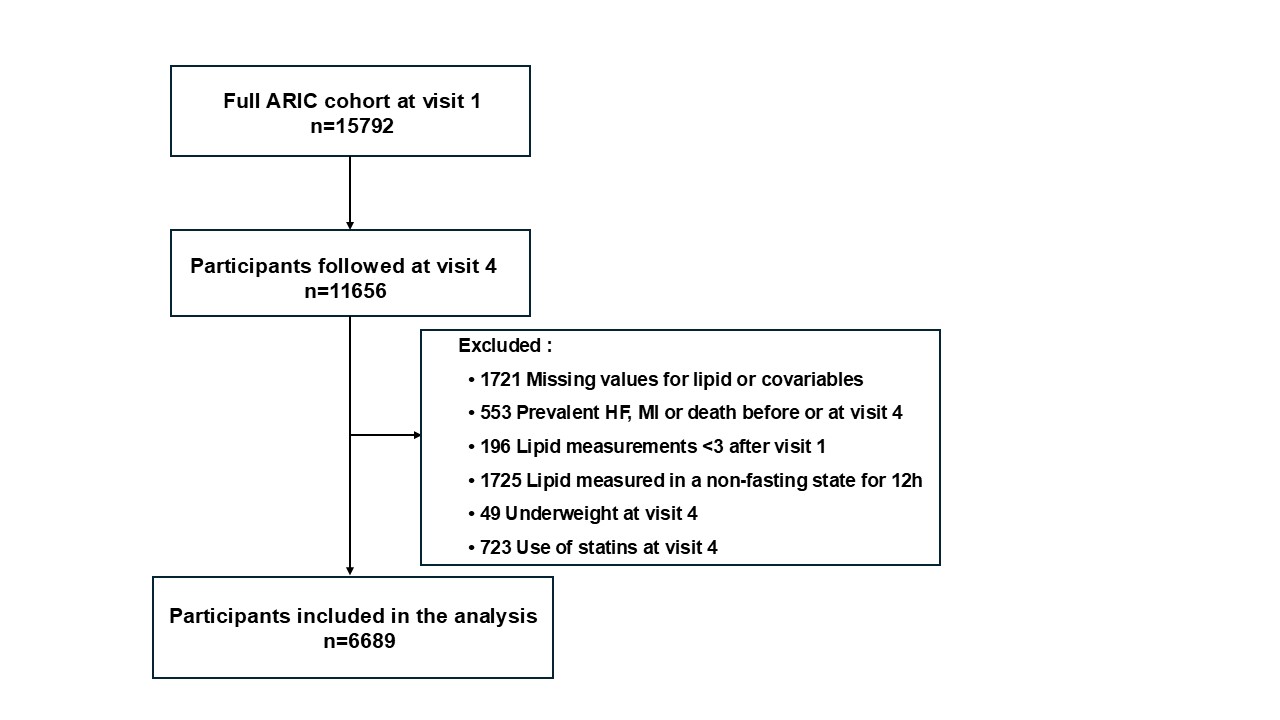


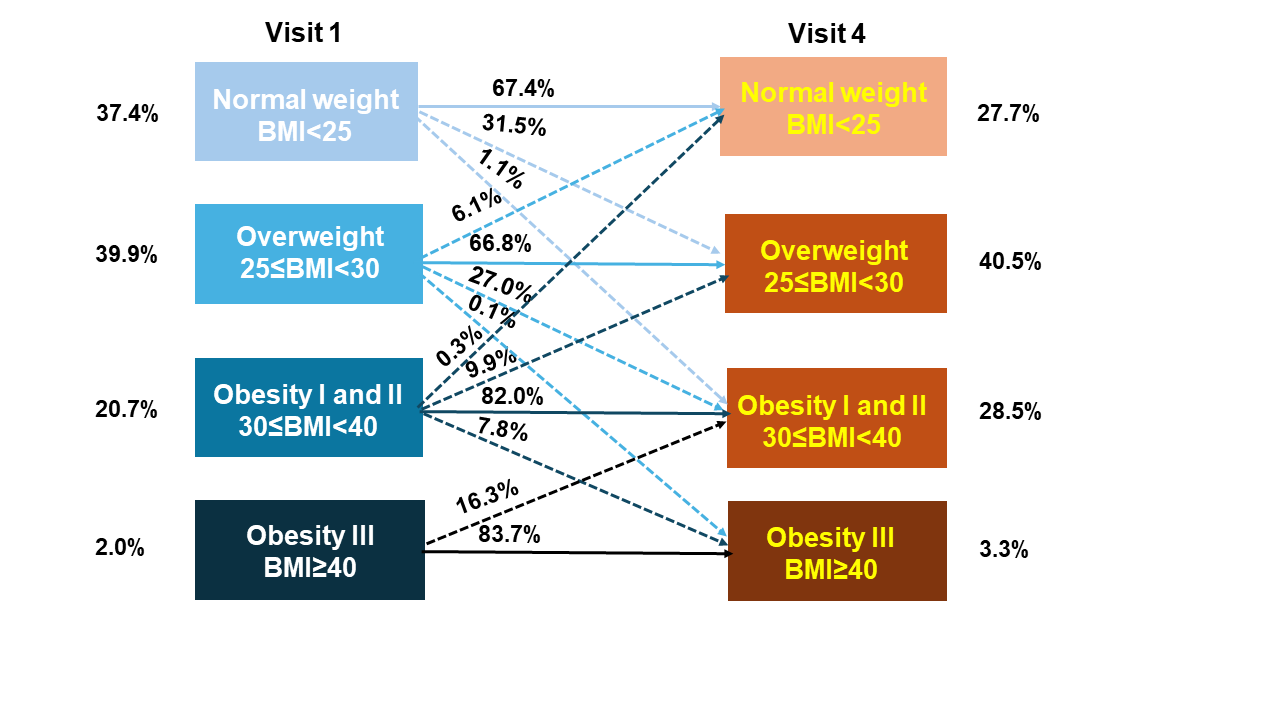


Supplementary FIGURE 1: Flow chart of study design. Abbreviations: HF indicates heart failure; MI, myocardial infarction.

Supplementary FIGURE 2: Change in body mass index (BMI) categories between ARIC visit 1 and visit 4. Solid arrows depict percent of adults at visit 1 who remained in their respective BMI category at visit 4. Dashed arrows indicate percent of participants with different BMI categories at visit 1 and visit 4.


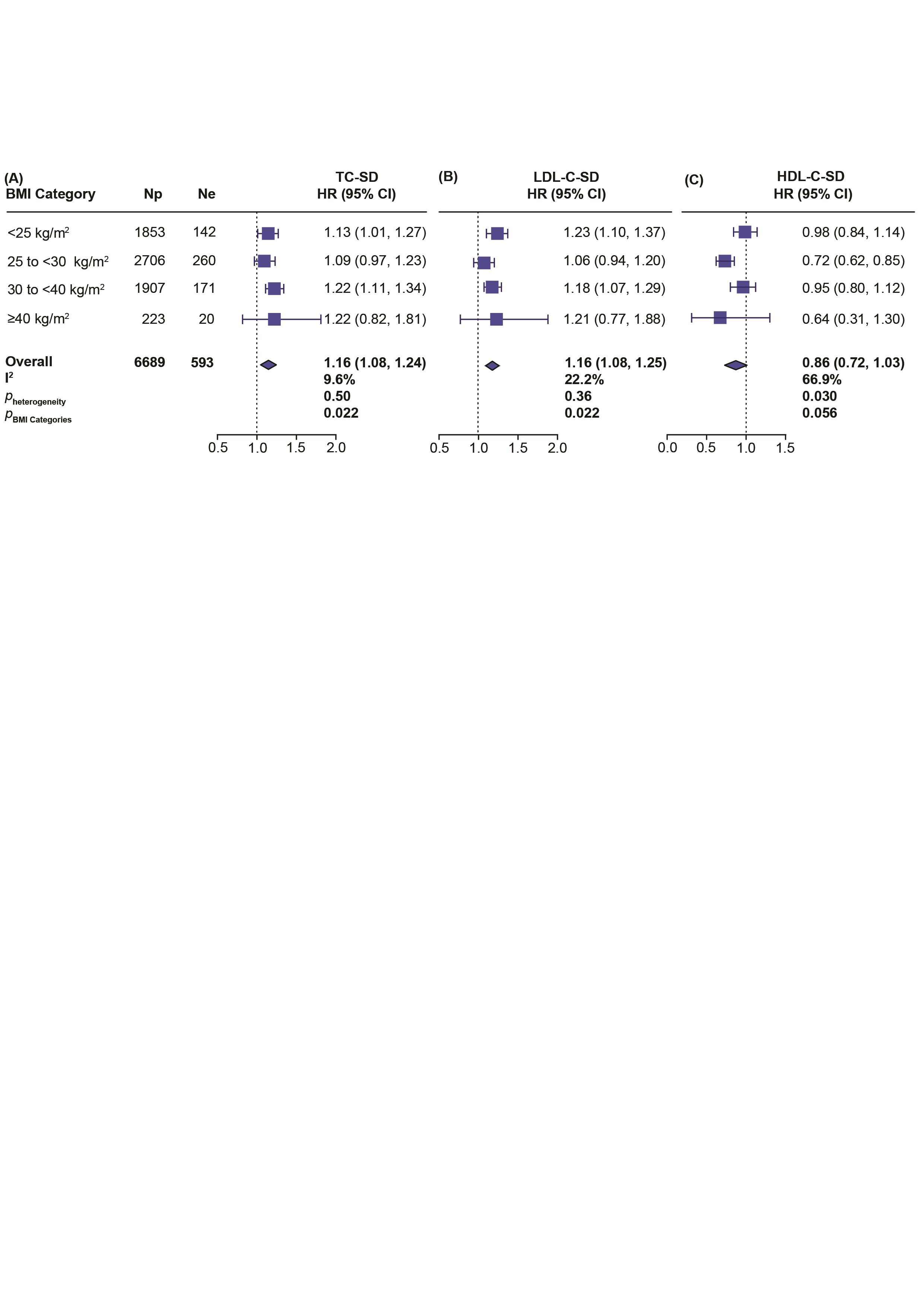


Supplementary FIGURE 3: The association of incident myocardial infarction with SD of total cholesterol (TC, A), LDL-C (B) and HDL-C (C) stratified by body mass index (BMI) category. Squares and horizontal lines represent the hazard ratio and 95% confidence interval for each survey cycle. Diamonds denote the pooled estimates with 95% confidence intervals. For I^2^, values < 25%, 25%–50%, and > 50% indicate modest, moderate, and substantial heterogeneity, respectively. *p*-values refer to the significance of hazard ratios over BMI category. Np number of participants at risk, and Ne the number of events.


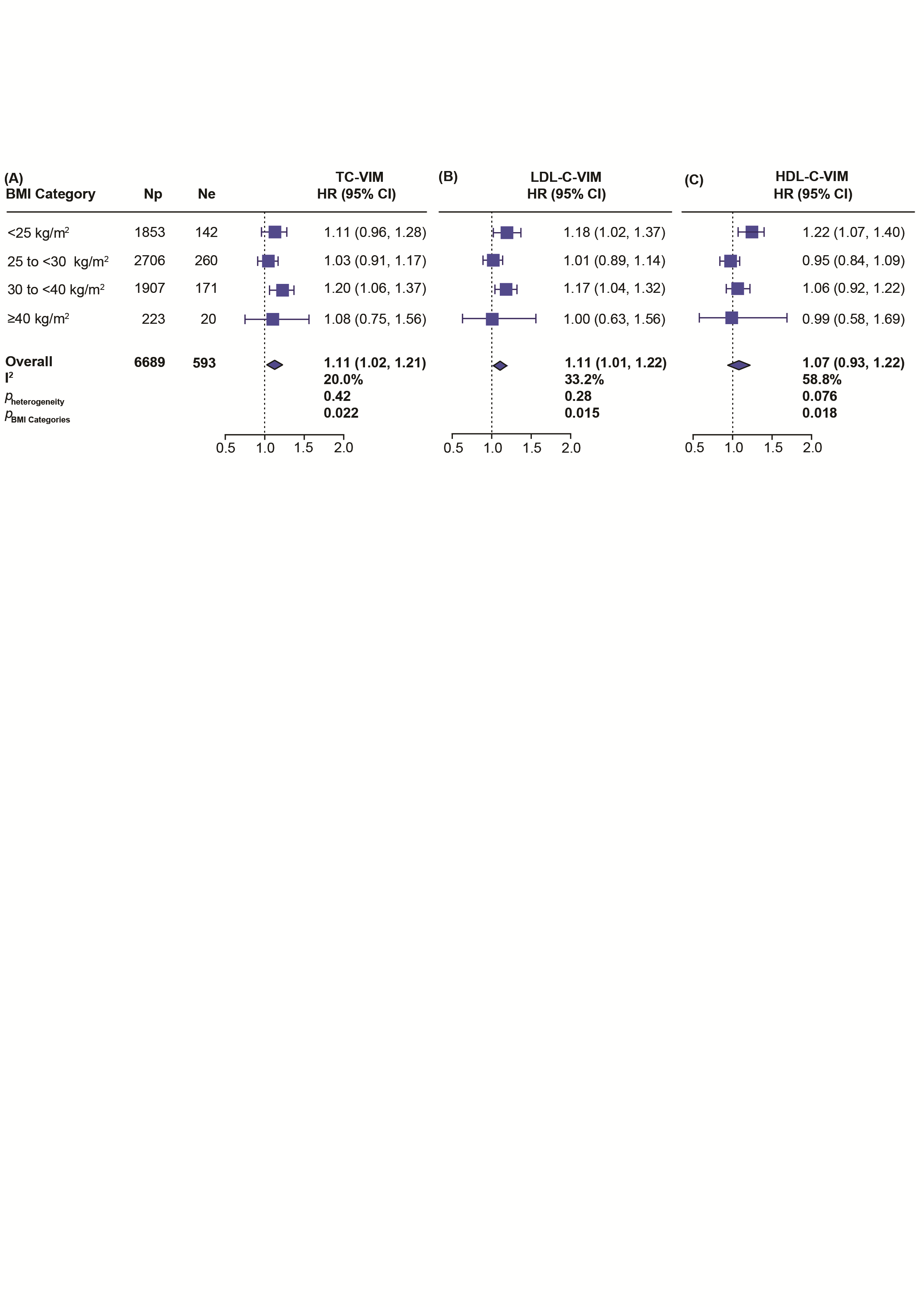


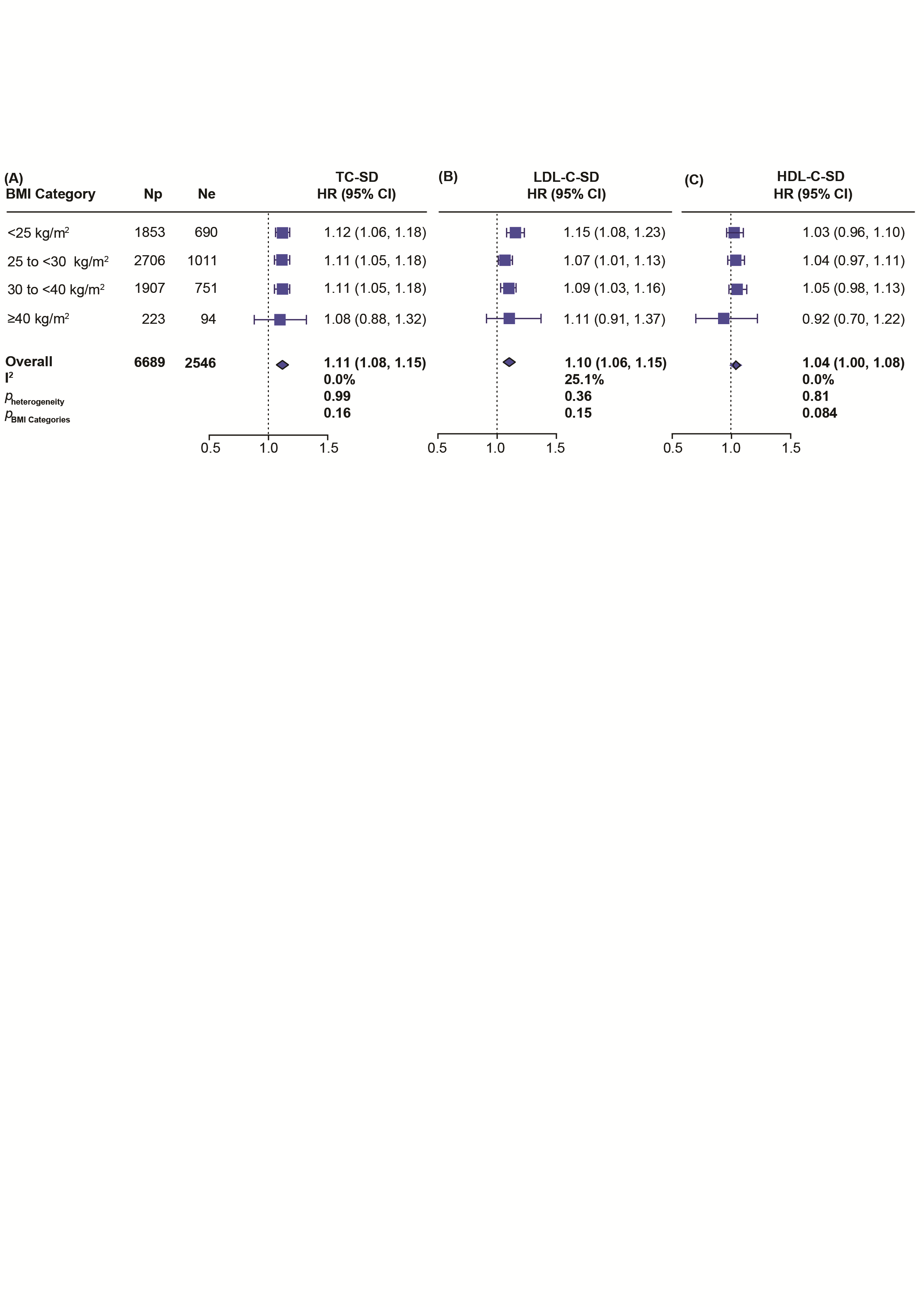


Supplementary FIGURE 4: The association of incident myocardial infarction with VIM of total cholesterol (TC, A), LDL-C (B) and HDL-C (C) stratified by body mass index (BMI) category. Squares and horizontal lines represent the hazard ratio and 95% confidence interval for each survey cycle. Diamonds denote the pooled estimates with 95% confidence intervals. For I^2^, values < 25%, 25%–50%, and > 50% indicate modest, moderate, and substantial heterogeneity, respectively. *p*-values refer to the significance of hazard ratios over BMI category. Np number of participants at risk, and Ne the number of events.

Supplementary FIGURE 5: The association of all‑cause mortality with SD of total cholesterol (TC, A), LDL-C (B) and HDL‑C (C) stratified by body mass index (BMI) category. Squares and horizontal lines represent the hazard ratio and 95% confidence interval for each survey cycle. Diamonds denote the pooled estimates with 95% confidence intervals. For I^2^, values < 25%, 25%–50%, and > 50% indicate modest, moderate, and substantial heterogeneity, respectively. *p*-values refer to the significance of hazard ratios over BMI category. Np number of participants at risk, and Ne the number of deaths.


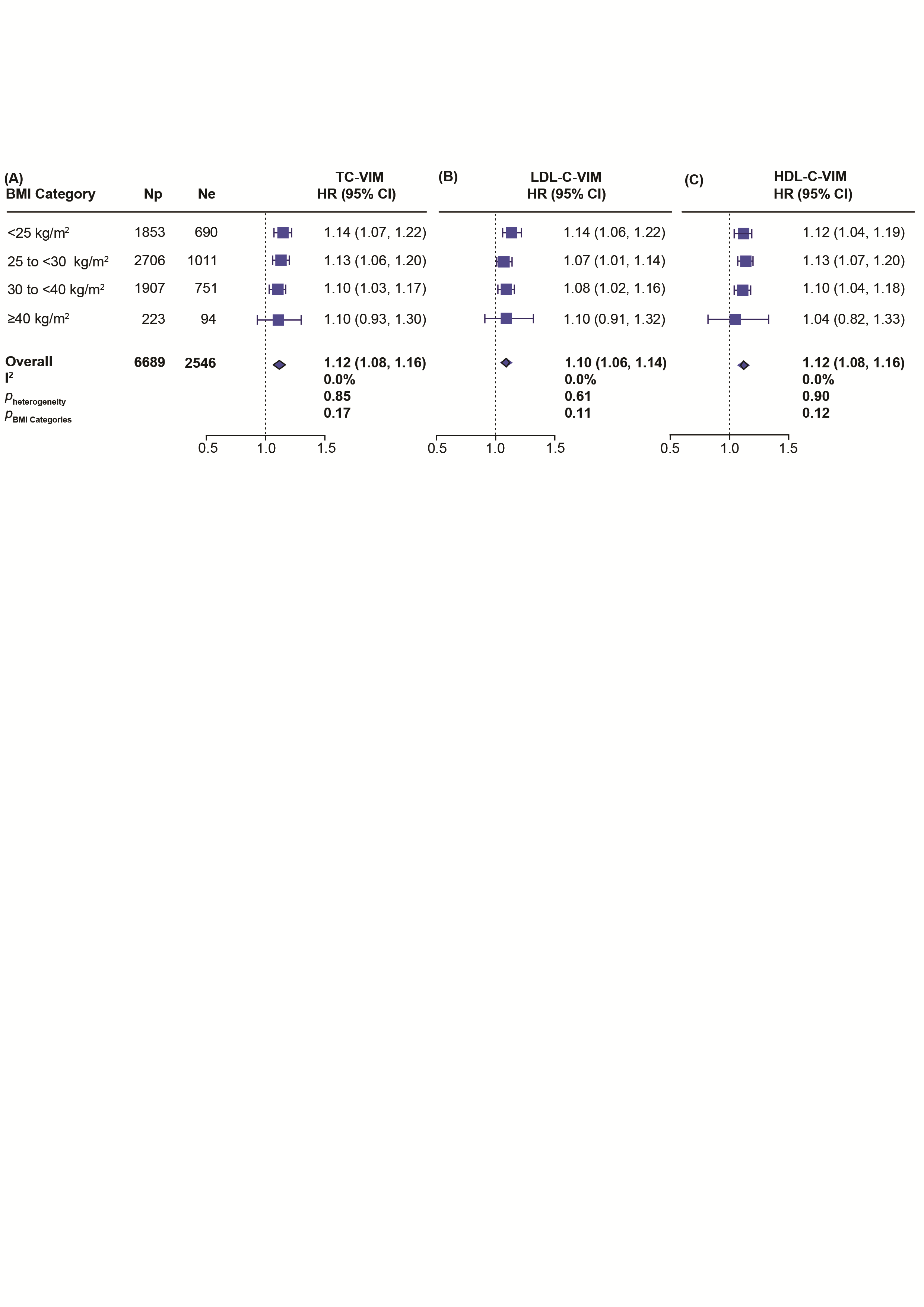


Supplementary FIGURE 6: The association of all‑cause mortality with VIM of total cholesterol (TC, A), LDL‑C (B) and HDL‑C (C) stratified by body mass index category. Squares and horizontal lines represent the hazard ratio and 95% confidence interval for each survey cycle. Diamonds denote the pooled estimates with 95% confidence. For I^2^, values < 25%, 25%–50%, and > 50% indicate modest, moderate, and substantial heterogeneity, respectively. *p*-values refer to the significance of hazard ratios over BMI category. Np number of participants at risk, and Ne the number of deaths.
